# Extracellular Vesicles from *Kluyveromyces marxianus* as Potential Postbiotics Against *Candida albicans* Vaginal Infections

**DOI:** 10.3390/pathogens15070667

**Published:** 2026-06-25

**Authors:** Marianna Imparato, Annalisa Buonanno, Angela Maione, Monica Matuozzo, Chiara D’Ambrosio, Andrea Scaloni, Marco Guida, Emilia Galdiero, Elisabetta de Alteriis

**Affiliations:** 1 Department of Biology, University of Naples “Federico II”, 80126 Naples, Italy; marianna.imparato@unina.it (M.I.); annalisa.buonanno@unina.it (A.B.); angela.maione@unina.it (A.M.); marco.guida@unina.it (M.G.); egaldier@unina.it (E.G.); 2 Proteomics, Metabolomics and Mass Spectrometry Laboratory, Istituto per il Sistema Produzione Animale in Ambiente Mediterraneo (ISPAAM), Consiglio Nazionale delle Ricerche, 80100 Napoli, Italy; monica.matuozzo@cnr.it (M.M.); chiara.dambrosio@cnr.it (C.D.); andrea.scaloni@cnr.it (A.S.); 3 BAT Center-Interuniversity Center for Studies on Bioinspired Agro-Environmental Technology, University of Naples “Federico II”, 80055 Portici, Italy; 4 National Biodiversity Future Center (NBFC), 90133 Palermo, Italy

**Keywords:** yeast, probiotics, biofilm, vulvo-vaginal candidiasis

## Abstract

This study describes extracellular vesicles (EVs) isolated from the culture supernatant of a *Kluyveromyces marxianus* strain deriving from an artisanal sourdough. Previous work had clearly shown the probiotic properties of the yeast isolate and its antagonistic activities against clinical fluconazole-resistant *Candida albicans* strains. Characterization of the isolated EVs by nanotracking particle analysis showed they had a mean diameter of 157.7 nm. Proteomic characterization of the purified EVs identified a complex array of 100 proteins. Both *C. albicans* planktonic growth and biofilm formation were inhibited by *K. marxianus* EVs, as well as adhesion and invasion of *Candida* cells in the vaginal epithelial A-431 cells. In the same cell model, *K. marxianus* EVs exerted an immunomodulatory effect affecting the secretion of pro-inflammatory and anti-inflammatory cytokines. Further, the expression of *C. albicans SAP2* and *SAP6* genes, coding for two aspartyl proteases involved in the invasion and damage of the epithelial mucosa, was affected by the presence of the yeast EVs. Overall, the results of this study show that *K. marxianus* EVs retain, at least in part, the beneficial features of the live microorganism, representing a postbiotic cell-free alternative preparation potentially useful for the management of *C. albicans* vaginal infections.

## 1. Introduction

The traditional focus on probiotics as live, beneficial microorganisms is now expanding to a deeper understanding of the components and metabolites they produce. This evolution has raised interest in the so-called “postbiotics”, defined as “preparations of inanimate microorganisms and/or their components that confers a health benefit on the host”, as stated by the International Scientific Association for Probiotics and Prebiotics (ISAPP) [1].

Extracellular vesicles (EVs) from probiotic microorganisms represent a specific and highly promising subclass of postbiotics. EVs are bilayered nanostructures secreted in the surrounding medium of all types of cells, including bacteria and fungi [2]. When produced from a probiotic microorganism, EVs can be recognized as a key mediator of health benefits and are being explored for their potential to provide similar protective and therapeutic effects while circumventing the limitations associated with live microorganisms [3]. Indeed, the widespread and increasing use of live probiotics has brought to light some concerns regarding their efficacy and safety. Their use has been associated with a high risk of infection and morbidity in neonates with very low birth weight; infant patients in intensive care units; and postoperative, hospitalized, or immunocompromised patients, in part due to bacteremia and fungemia [4,5]. Most of the literature refers to EVs from probiotic lactic acid bacteria (LAB) [6,7,8]. Functional capabilities of such EVs have been explored in a variety of disease contexts [9], with promising results from in vitro and in vivo models, though clinical trials are still lacking. EVs have been especially considered to treat gastrointestinal disorders, since they have been found to restore gut microbiota dysbiosis and maintain the integrity of the intestinal epithelial barrier [10]. EVs have also been reported to be implicated in the gut–brain axis, mediating cellular communication in the central nervous system [11].

In the context of infectious diseases, EVs have been used for the development of EV-based vaccines more than as potential anti-infective entities [12,13]. The protective capacity of EVs against pathogen infections was only studied against one virus (HIV-1) and a few bacteria (*Staphylococcus aureus*, *Salmonella typhimurium*, and *Escherichia coli*) [14,15,16], and there is still no information on the effect against fungal and parasitic infections [13].

Unlike EVs from probiotic bacteria, research on EVs from probiotic yeasts is still in its infancy. Instead, EV morphology and content have been analyzed for several pathogenic yeast species, with a major part of the research being focused on the interactions of the pathogenic fungi with the host [17,18]. A few recent reports have evidenced interesting features of EVs from non-pathogenic yeasts, sometimes assessed as probiotic yeasts, revealing their immunomodulation potential [19,20] and the ability to transfer their cargo molecules into the host cells [21]. In some cases, the proteomic content of the produced EVs has been investigated [19,22,23]. Recently, evidence for the effects of EVs isolated from the probiotic yeast *Pichia kudriavzevii* against *Salmonella* has been reported [24].

Fermented foods and beverages are a potential source for new yeast isolates showing probiotic features [25,26]. In this context, we have recently isolated from an artisanal sourdough a new yeast strain that was identified as *Kluyveromyces marxianus* [27]. *K*. *marxianus* is a non-conventional food-grade yeast, known for its wide biotechnological applications [27,28]. We assessed the new isolate for its probiotic and safety properties, which produced comparable results to those exhibited by the commercial probiotic *Saccharomyces boulardii* [29]. In the same study, we demonstrated the antagonistic activity of the isolate against some clinical fluconazole-resistant *Candida albicans* strains. Since *C. albicans* is the major responsible agent for vulvo-vaginal candidiasis (VVC) [30], the protective effect of the novel isolate demonstrated the biotherapeutic potential of this food-grade yeast in the prevention and treatment of such pathology [29].

Here, we have hypothesized that the anti-*Candida* effects displayed by *K. marxianus* could be associated with the EVs secreted by the yeast into the culture medium. Therefore, we isolated such EVs from the culture supernatant of the *K. marxianus* isolate and characterized them, especially focusing on their proteomic content. Once the safety of *K. marxianus* EVs was assessed in vitro and in vivo, we explored their antagonistic activity against *C. albicans*, specifically concerning the inhibition of growth and the formation of biofilm. Using a vaginal epithelial cell model represented by A-431 cells, we also investigated the inhibitory effects of the isolated EVs on both adhesion and invasion of *Candida* cells, as well as their immunomodulatory effect by determining the secretion of pro-inflammatory and anti-inflammatory cytokines. The effect of the probiotic EVs was also extended to the expression of two *Candida* genes, *SAP2* and *SAP6*, coding for two aspartyl proteases involved in the invasion and damage of the epithelial mucosa [31].

## 2. Materials and Methods

### 2.1. Strains and Culture Conditions

The *Kluyveromyces marxianus* strain used in this work was isolated from a home-made sourdough, as previously reported [29]. It was maintained in YPD agar: 1% *w*/*v* yeast extract, 2% *w*/*v* bacto peptone (Gibco, Life Technologies Co., Detroit, MI, USA), 2% *w*/*v* dextrose, and 2% *w*/*v* agar (Sigma Aldrich, St. Louis, MO, USA). Pre-cultures and cultures of *K. marxianus* were performed in shake flasks containing YPD medium, starting from a single colony, and incubated at 30 °C for 24 h, at 200 rpm. The strain of *C. albicans* used in this work was a clinical fluconazole-resistant one, named C19, and isolated from VVC patients [32]. It was considered representative of a series of fluconazole-resistant strains that have already shown similar responses to the action of live *K. marxianus* cells, as already reported [29]. C19 strain was cultured in YPD medium at 37 °C.

### 2.2. EV Isolation and Characterization

EVs were isolated from the supernatants of *K. marxianus* cultures grown in YPD medium for 24 h. Initially, culture supernatants were cleared of cells and debris by centrifugation at 8000 *g* for 20 min at 4 °C. The resulting cell-free supernatants were then concentrated using Amicon ultrafiltration devices with a 100 kDa molecular weight cut-off (Merck, Darmstadt, Germany), followed by filtration through Ultrafree-CL centrifugal filters with a pore size of 0.45 µm (Sigma-Aldrich). To verify the complete removal of fungal cells, aliquots of the filtered supernatants were plated onto YPD agar and incubated to monitor yeast growth. The concentrated and filtered supernatants were subsequently subjected to ultracentrifugation at 100,000 *g* for 1 h at 4 °C. The resulting pellets were washed once in sterile PBS by repeating the ultracentrifugation step under the same conditions. Final EV pellets were resuspended in 100 µL of sterile PBS. All ultracentrifugation steps were performed using an Optima MAX-XP ultracentrifuge equipped with a TLA 100.3 rotor (Beckman Coulter, Brea, CA, USA). The isolated EVs were stored at −80 °C until further analysis.

EV characterization was performed using nanoparticle tracking analysis (NTA). EV size distribution and concentration were measured with the Nanosight NS300 platform (Malvern Panalytical, Malvern, UK) equipped with a sCMOS camera and a green laser, operating under the control of NTA software v. 3.4. Samples were diluted 1:100 in PBS and analyzed with a camera level set to 16 and a detection threshold of 4. Videos were recorded to visualize EVs in each sample. Dynamic light scattering (DLS) was used to measure the zeta potential of EVs with a Zetasizer Nano-ZS (Malvern Instruments, Worcestershire, UK). Samples were prepared at a concentration of X in PBS. Measurements were carried out at 25 °C using a 4 mW He–Ne laser (633 nm) with a fixed scattering angle of 173°.

### 2.3. Protein Extraction, Separation, and Digestion

Protein extraction was performed on two biological replicates of *K. marxianus* EVs. The samples were lysed in parallel using a buffer composed of 8 M urea and 1% *w*/*v* SDS, supplemented with a complete EDTA-free protease inhibitor cocktail (Roche, Mannheim, Germany) at a 1:10 *v*/*v* ratio. To ensure maximal disruption, EVs were first subjected to probe sonication (0.3 cycles) on ice for 2 min, followed by two 2 min rounds of bath sonication. After an additional 30 min of incubation on ice, the lysates were clarified by centrifugation at 6000 *g* for 15 min at 4 °C. Finally, protein concentration was determined using the Pierce BCA Protein Assay kit (Thermo Fisher Scientific, San Jose, CA, USA) following the manufacturer’s instructions. Finally, proteins were precipitated with 6 vol of cold acetone overnight at −20 °C. After precipitation, proteins were pelleted by centrifugation at 8000 *g* for 20 min at 4 °C and then vacuum-dried with a SpeedVac roto-evaporator (Thermo Fisher Scientific). Recovered proteins were separated by 12% T SDS-PAGE under reducing conditions. Following colloidal Coomassie Blue staining and destaining, each gel lane was excised into 15 slices. These slices were triturated and washed with water and acetonitrile, followed by protein reduction with 10 mM dithiothreitol and alkylation with 55 mM iodoacetamide (both in 100 mM NH_4_HCO_3_). In-gel digestion was then performed using trypsin (12.5 ng/µL in 50 mM NH_4_HCO_3_). The resulting peptide mixtures were purified and concentrated using ZipTip μC18 (Millipore, Burlington, MA, USA), vacuum-dried, and finally resuspended in 5% *v*/*v* formic acid.

#### 2.3.1. Mass Spectrometry

Peptide mixtures were characterized by a nanoUltraHighPressure liquid chromatography (nUHPLC)-electrospray (ESI)-Q-Orbitrap-MS/MS platform consisting of a Vanquish-Neo nano-chromatographer (Thermo Fisher Scientific) linked to an Exploris 480 mass spectrometer (Thermo Fisher Scientific) through an easy-spray ion source. Peptides were loaded on an EASY-Spray C18 column (250 mm × 75 μm ID, 2 μm particles, 100 Å pore size) (Thermo Fisher Scientific) and eluted with a gradient of solvent B (19.92/80/0.08 *v*/*v*/*v* water/acetonitrile/formic acid) in solvent A (99.9/0.1 *v*/*v* water/formic acid) at a flow rate of 250 nL/min. The gradient of solvent B started at 6%, increased to 31% over 90 min, increased to 50% over 5 min, increased to 95% over 5 min, remained at 95% for 8 min, and finally returned to 6% for the equilibrating step. The mass spectrometer operated in data-dependent acquisition mode collecting MS spectra of positive ions within a scan *m*/*z* range of 375–1200, using a nominal resolution of 120,000 full width at half maximum (FWHM), an automatic gain control (AGC) target of 3 × 10^6^ ions, a maximum injection time (IT) of 50 ms, and a dynamic exclusion value of 45 s, which was followed by MS/MS scans of the 20 most abundant ions. MS/MS spectra were acquired using a normalized collision energy (HCD) of 30%, a resolution of 15,000 FWHM, an AGC target of 2 × 10^5^ ions, and a maximum IT of 100 ms.

#### 2.3.2. Bioinformatics

For protein identification, raw mass spectrometry data were processed using Proteome Discoverer software (v. 3.1, Thermo Fisher Scientific, San Jose, CA, USA). Database searching was performed by the Mascot algorithm (v. 2.4.2, Matrix Science, London, UK) against the *K. marxianus* UniProtKB sequence database (48,266 protein entries), which included common protein contaminants. The search parameters were configured as follows: trypsin was set as the proteolytic enzyme with a maximum of two missed cleavages; carbamidomethylation of cysteine was specified as a fixed modification, and oxidation of methionine, deamidation of asparagine and glutamine, and pyroglutamate formation at N-terminal glutamine were included as variable modifications [33]. Precursor and fragment mass tolerances were set to ±10 ppm and ±0.02 Da, respectively. Confidence criteria for protein identification required at least one unique peptide, two peptide–spectrum matches (PSMs), and a Mascot score ≥ 25. All assignments were further validated by manual spectra inspection and filtered to a 1% false discovery rate (FDR).

Proteomic data were deposited in the ProteomeXchange Consortium [34]. The proteomic dataset has been deposited in the PRIDE repository [35] under the identifier PXD078607 and is publicly available at the following URL: https://www.ebi.ac.uk/pride/archive/projects/PXD078607/public) (accessed 20 May 2026). Identified *K. marxianus* vesicle proteins were associated with components of EVs already assigned to other organisms according to the literature. Functional relationships among the identified *K. marxianus* vesicle proteins were analyzed and visualized using STRING software (v. 12.0). Functional enrichment, subcellular localization, and biological pathway analyses were performed using integrated STRING resources, including Gene Ontology (GO) [36], COMPARTMENTS [37], and Reactome (version 12.0) [38] software.

### 2.4. A-431 Cells

The human epithelial A-431 cell line derived from a vaginal epithelial squamous cell carcinoma was used. The cell line was purchased from DSZM (Braunschweig, Germany). The cells were cultured in Dulbecco’s modified Eagle medium (DMEM; Sigma Aldrich) supplemented with L-glutamine (2 mM) (Sigma Aldrich), 1% *w*/*v* penicillin–streptomycin (Sigma Aldrich), and a heat-inactivated Fetal Bovine Serum (FBS) at 10% *w*/*v* (Sigma Aldrich); specifically, the cell line was kept in culture by-passages in fresh medium twice a week and incubated in a 5% CO_2_ atmosphere at 37 °C.

### 2.5. Evaluation of EV Safety

The in vitro cytotoxicity of *K. marxianus* EVs on the A-431 cell line was evaluated using both the MTT assay and the determination of lactic dehydrogenase (LDH) release, an indicator of epithelial cell damage. Cells were seeded at a density of 2 × 10^5^ cells/well in 96-well plates and incubated to allow adhesion overnight. After 24 h, when cells were at 80% confluency, EVs were added at concentrations of 10^5^, 10^6^, 10^7^, 10^8^, and 10^9^ EVs/mL. After a 24 h exposure period, the MTT reagent (Sigma Aldrich) was added and incubated for 3–4 h. The resulting formazan crystals were dissolved, and absorbance was measured at 570 nm using a Varioskan LUX Multimode Microplate Reader (Thermo Fisher Scientific, USA). Cell viability percentages were calculated relative to untreated control cells.

In parallel, culture supernatants were collected, and LDH levels were quantified using a colorimetric assay kit (Sigma Aldrich), following the manufacturer’s instructions. Absorbance was read at 490 nm using the microplate reader reported above.

The in vivo safety of *K. marxianus* EVs was assessed using *Galleria mellonella* larvae. Seven experimental groups of larvae were used, including five groups of EV-injected larvae—from 10^6^ to 10^10^ EVs suspended in sterile PBS per larva—and two control groups. The two control groups consisted of intact (non-injected) larvae and PBS-injected larvae. For each experimental group, ten randomly selected larvae at a comparable developmental stage were used. A Hamilton syringe was used to inject 10 µL of sample into the left hind proleg of each larva. Larval survival was monitored at 37 °C over a 3-day period. The experiment was conducted in three independent biological replicates.

### 2.6. Antagonistic Activity of EVs in Liquid Culture

To assess the antagonistic activity of *K. marxianus* EVs in liquid culture, *C. albicans* C19 was allowed to grow in wells of a 96-well microplate, containing 100 mL of YPD medium with or without yeast EVs at two densities: 6 × 10^9^ and 1.2 × 10^10^ EVs/mL. *C. albicans* C19 growth started with an inoculum corresponding to OD_590_ = 0.1. The microplate was incubated at 30 °C, and the increase in OD_590_ was monitored for 18 h using the microplate reader reported above.

### 2.7. Inhibition of Biofilm Formation

For the development of biofilms in static conditions, *C. albicans* C19 cells, derived from an overnight pre-culture in YPD, were adjusted to 10^6^ CFU/mL with the YPD culture medium, in the presence or absence of *K. marxianus* EVs at two densities (6 × 10^9^ and 1.2 × 10^10^ EVs/mL). Then, 100 μL of each culture was pipetted into wells of a polystyrene 96-well microplate. Biofilms were allowed to develop for 24 h at 37 °C in static conditions. Quantification of total biofilm biomass was performed with crystal violet staining, as previously described [39]. The percentage of biofilm inhibition was calculated with respect to the reduction in OD_470_ of the control and was calculated as follows: % biofilm inhibition = (OD_470_ control − OD_470_ sample/OD_470_ control) × 100.

### 2.8. Adhesion and Invasiveness Assays

The ability of *K. marxianus* EVs to modulate the adhesion and invasiveness of C19 was evaluated in A-431 cells. They were seeded at a density of 2.5 × 10^5^ cells/mL in DMEM and, after 24 h of incubation to allow cell monolayer formation at 37 °C in a 5% CO_2_ atmosphere, they were exposed to EVs at a density of 10^9^ EVs/mL for 2 h. Subsequently, C19 was added at a multiplicity of infection (MOI) of 1:100 and incubated for an additional 2 h under the same conditions. At the end of this period, non-adherent yeast cells were removed by washing the monolayers three times with sterile PBS. To evaluate adhesion, A-431 cells were detached with 0.25% *w*/*v* trypsin, and the resulting samples were serially diluted and plated on YPD agar plates. Plates were incubated at 37 °C overnight, and colony-forming units of *Candida* (CFUs/mL) were counted to quantify the total cell-associated yeasts.

To assess *Candida* C19 invasion, after the initial infection phase and PBS washes, the monolayers were incubated for an additional 2 h in DMEM supplemented with Amphotericin B (5 µg/mL) (Sigma-Aldrich) to eliminate adhered yeast cells. The A-431 cells were then lysed with 100 µL of X% *v*/*v* Triton X-100 and processed as described above to quantify the internalized *Candida* cells (CFUs/mL).

### 2.9. Quantification of Cytokines by ELISA

To evaluate the immunomodulatory effect of *K. marxianus* EVs, the production of some cytokines was evaluated by pre-treating cells with EVs before infection. Aiming at this, A-431 cells were seeded in a 12-well plate and incubated for 24 h. The following day, after reaching a 80% confluence, the cells were pre-treated with EVs alone (10^8^ vesicles/mL) and, after 2 h incubation, infected with C19 (MOI 1:100).

Untreated cells served as the control group to evaluate the effect of the infection alone on the cytokine production.

After 24 h, culture supernatants were collected for the quantification of cytokine levels. ELISA kits (Elabscience, Wuhan Elabscience Biotechnology Co., Wuhan, China) were used to measure the concentrations of IL-6, IL-4, IL-10, IL-8, and IL-1β, following the manufacturer’s instructions. Cytokine concentrations in the test samples were evaluated with reference to standard curves.

### 2.10. qRTPCR (SAP1, SAP2)

To check the effect of *K. marxianus* EVs on the expression of the *SAP2* and *SAP6* genes of *C. albicans*, aliquots of *C. albicans* C19 strain (10^6^ cells/mL) were incubated in a YPD medium or a YPD medium plus 1% *v*/*v* of the bovine serum albumin (Sigma) for 24 h at 37 °C under agitation (150 rpm) in the presence or absence of yeast EVs, with the latter at a concentration of 1.6 × 10^8^ particles/mL. Primers for *SAP2* and *SAP6* were the same as previously described [29]. At the end of incubation, yeast cells were collected by centrifugation (5000 *g*, 10 min), washed with PBS, and the total RNA was extracted using the Direct-zolTM RNAMiniprep Plus Kit (ZYMO RESEARCH, Irvine, CA, USA). Then, 1 μg of the RNA was reverse transcribed to cDNA (Bio-Rad, Milan, Italy) and analyzed by a quantitative PCR run on a AriaMx Real-Time PCR instrument (Agilent Technologies, Inc., Milan, Italy), according to the manufacturer’s instructions. For the real-time PCR reaction, 100 ng of cDNA was used. The PCR system setup was as follows: 95 °C for 10 min, one cycle for cDNA denaturation; 95 °C for 15 s and 60 °C for 1 min, 40 cycles for amplification; 95 °C for 15 s, one cycle for final elongation; and one cycle for melting curve analysis (from 60 to 95 °C) to verify the presence of a single product. The expression levels of genes were evaluated using the comparative Ct method (2^−ΔΔCt^ method), with *ACT1* used as the reference gene for data normalization. The results were expressed as relative fold change compared with the untreated control condition.

### 2.11. Statistical Analysis

Statistical analyses were performed using GraphPad Prism software (version 8.02 for Windows; GraphPad Software, La Jolla, CA, USA; www.graphpad.com). The results are expressed as the mean ± standard deviation (SD) of at least three independent experiments. Survival curves were analyzed using the Kaplan–Meier method. Comparisons between groups were performed using one-way ANOVA followed by Dunnett’s post hoc test. A *p*-value of less than 0.05 was considered statistically significant.

## 3. Results

### 3.1. Isolation and Characterization of EVs from the Probiotic Yeast K. marxianus

**Figure 1:**
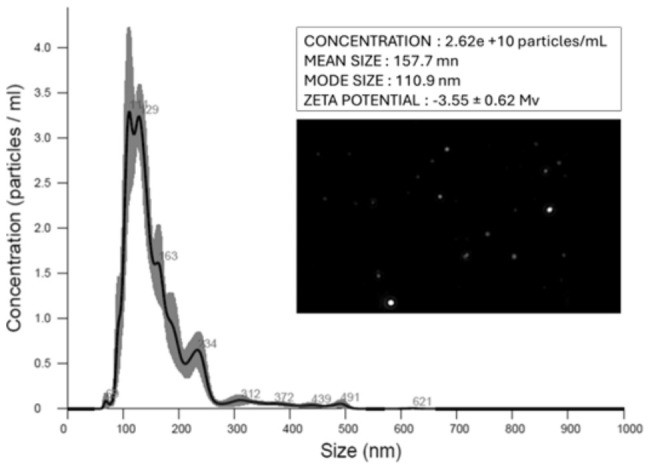
Size profile and particle concentrations of *K. marxianus* EVs determined by NTA as reported in the experimental section. The zeta potential of EVs was measured by DLS as described in the experimental section.

Following the isolation of *K. marxianus* EVs from the yeast supernatants of cultures, as described in the experimental section, the resuspended pellets were analyzed by nanoparticle tracking analysis (NTA). The analysis clearly showed the occurrence of EVs in the sample at an estimated concentration of 2.5 × 10^10^ particles/mL and with a mean diameter of 157.7 nm. The zeta potential value of the isolated EVs measured by DLS was −3.55 ± 0.62 mV (Figure 1).

### 3.2. Proteomic Analysis

**Figure 2:**
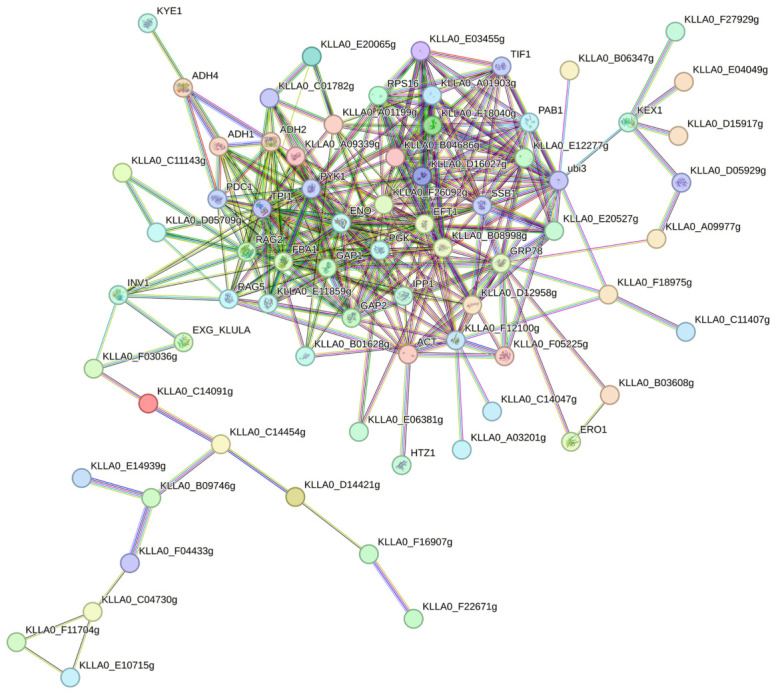
Protein–protein interaction (PPI) network of proteins identified in *K. marxianus* EVs using STRING software. The network shows functional protein associations assigned with a medium-confidence threshold (0.400). ACT: actin; ADH1: alcohol dehydrogenase 1; ADH2: alcohol dehydrogenase 2; ADH4: alcohol dehydrogenase 4, EFT1: elongation factor 2; ENO: enolase; ERO1: endoplasmic reticulum oxidoreductin-1; EXG_KLULA: glucan 1,3-beta-glucosidase; FBA1: fructose-bisphosphate aldolase; GAP1: glyceraldehyde-3-phosphate dehydrogenase 1; GAP2: glyceraldehyde-3-phosphate dehydrogenase 2; GRP78: endoplasmic reticulum chaperone BiP; HTZ1: histone H2A.Z; INV1: invertase; IPP1: inorganic pyrophosphatase; KEX1: protease KEX1; KLLA0_A01001g: KLLA0A01001p; KLLA0_A01199g: adenosylhomocysteinase; KLLA0_A01903g: KLLA0A01903p member of the universal ribosomal protein uL11 family; KLLA0_A03201g: KLLA0A03201p member of the glycosyl hydrolase 17 family; KLLA0_A03377g: KLLA0A03377p; KLLA0_A06468g: KLLA0A06468p; KLLA0_A06556g: KLLA0A06556p; KLLA0_A09339g: 6-phosphogluconate dehydrogenase; KLLA0_A09977g: carboxypeptidase; KLLA0_B01628g: KLLA0B01628p; KLLA0_B03608g: alpha-1,2-mannosidase; KLLA0_B04686g: KLLA0B04686p; KLLA0_B06347g: KLLA0B06347p; KLLA0_B07370g: KLLA0B07370p; KLLA0_B07447g: KLLA0B07447p; KLLA0_B08998g: elongation factor 1-alpha; KLLA0_B09746g: KLLA0B09746p; KLLA0_B14322g: KLLA0B14322p; KLLA0_C00572g: KLLA0C00572p; KLLA0_C01496g: KLLA0C01496p member of the CRISP family; KLLA0_C01782g: S-adenosylmethionine synthase; KLLA0_C04730g: KLLA0C04730p member of the glycosyl hydrolase 18 family; KLLA0_C07238g: 1,3-beta-glucanosyltransferase; KLLA0_C11143g: KLLA0C11143p; KLLA0_C11407g: KLLA0C11407p; KLLA0_C14047g: KLLA0C14047p member of the glycosyl hydrolase 17 family; KLLA0_C14091g: 1,3-beta-glucanosyltransferase; KLLA0_C14454g: KLLA0C14454p; KLLA0_C17985g: KLLA0C17985p; KLLA0_C19338g: KLLA0C19338p member of the histidine acid phosphatase family; KLLA0_C19437g: KLLA0C19437p; KLLA0_D02442g: KLLA0D02442p member of the CRISP family; KLLA0_D05709g: phosphomannomutase; KLLA0_D05863g: KLLA0D05863p; KLLA0_D05929g: KLLA0D05929p member of the peptidase A1 family; KLLA0_D12958g: KLLA0D12958p; KLLA0_D14421g: 1,3-beta-glucanosyltransferase; KLLA0_D15917g: KLLA0D15917p member of the peptidase A1 family; KLLA0_D16027g: KLLA0D16027p; KLLA0_E01299g: KLLA0E01299p; KLLA0_E02047g: KLLA0E02047p; KLLA0_E03455g: KLLA0E03455p; KLLA0_E04049g: KLLA0E04049p member of the peptidase A1 family; KLLA0_E04995g: KLLA0E04995p; KLLA0_E06381g: KLLA0E06381p; KLLA0_E10715g: KLLA0E10715p; KLLA0_E11859g: phosphoglycerate mutase; KLLA0_E12277g: KLLA0E12277p; KLLA0_E14939g: KLLA0E14939p; KLLA0_E20065g: KLLA0E20065p; KLLA0_E20527g: KLLA0E20527p member of the heat shock protein 70 family; KLLA0_E24861g: KLLA0E24861p; KLLA0_E24927g: KLLA0E24927p; KLLA0_F01012g: KLLA0F01012p; KLLA0_F03036g: KLLA0F03036p member of the glycosyl hydrolase 17 family; KLLA0_F04433g: KLLA0F04433p; KLLA0_F05225g: KLLA0F05225p member of the small GTPase superfamily; KLLA0_F11704g: KLLA0F11704p; KLLA0_F12100g: KLLA0F12100p member of the 14-3-3 family; KLLA0_F16907g: glycosidase; KLLA0_F18040g: KLLA0F18040p member of the universal ribosomal protein uS15 family; KLLA0_F18975g: KLLA0F18975p; KLLA0_F22671g: glycosidase; KLLA0_F26092g: KLLA0F26092p; KLLA0_F27929g: KLLA0F27929p; KYE1: enoatereductase1; PAB1: polyadenylate-binding protein, PDC1: pyruvate decarboxylase; PGK: phosphoglycerate kinase; PLB: lysophospholipase; PYK1: pyruvate kinase; RAG2: glucose-6-phosphate isomerase; RAG5: hexokinase; RPS16: 40S ribosomal protein S16; SSB1: ribosome-associated molecular chaperone SSB1; TIF1: ATP-dependent RNA helicase eIF4A; TPI1: triosephosphate isomerase; ubi3: ubiquitin-40S ribosomal protein S27a.

To characterize the protein cargo of *K. marxianus* EVs, a comprehensive proteomic analysis was performed. Briefly, vesicle proteins were extracted and resolved by SDS-PAGE, followed by in-gel trypsin digestion. The resulting peptides were analyzed by nUHPLC-ESI-Q-Orbitrap-MS/MS; subsequent bioinformatic analysis of the MS data allowed the identification of a total of 100 *K. marxianus* proteins (Supplementary Table S1). Based on literature reports, several of these proteins (76 in number, corresponding to 76% of the whole assigned molecules) were recognized as components of the protein cargo of EVs from other microorganisms, such as *Candida albicans* [40], *Candida auris* [41], *Saccharomyces cerevisiae* [22,42], *Cryptococcus neoformans*, and *Cryptococcus deuterogattii* [43]. To elucidate the functional landscape of the *K. marxianus* vesicular proteome, a protein–protein interaction (PPI) network was constructed using the STRING database (Figure 2). A medium-confidence threshold (0.400) was applied to ensure a comprehensive yet representative set of functional associations. The resulting network, comprising 94 proteins, revealed a highly interconnected architecture. Topologically, the right cluster of the network exhibits a dense web of reciprocal interactions, identifying a central functional core likely involved in tightly integrated cellular activities. In contrast, the left portion displays a more linear and sparser topology with sequential connections, characteristics of peripheral sub-modules or specialized pathways. The biological relevance of this organization was supported by a PPI enrichment *p*-value < 1 × 10^−16^, confirming that the observed connectivity is significantly higher than would be expected by chance.

**Figure 3:**
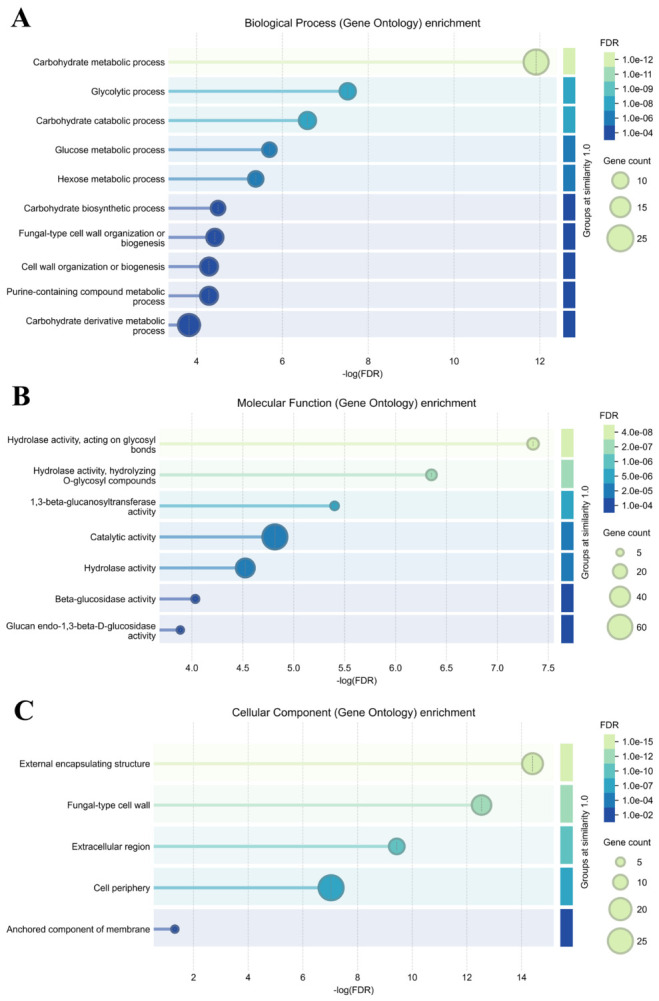
Enriched Biological Process (BP) (**A**), Molecular Function (MF) (**B**), and Cellular Component (CC) (**C**) Gene Ontology terms associated with proteins identified in *K. marxianus* EVs. The figure shows the most significantly enriched BP, MF, and CC terms based on false discovery rate (FDR) (color scale) and number of associated genes (bubble dimension).

Furthermore, a functional enrichment analysis was performed on *K. marxianus* vesicle proteins, focusing on Gene Ontology (GO) categories, including Biological Process (BP), Molecular Function (MF), and Cellular Component (CC) (Figure 3). Regarding the BP-GO category (Figure 3A), a pronounced dominance of central carbohydrate metabolism was observed. Processes such as glycolysis and hexose catabolism were characterized by the highest statistical significance and gene counts, thereby identifying them as the primary metabolic drivers within the dataset. This metabolic orientation is found to be closely coordinated with fungal-type cell wall organization and biogenesis. Similarly, in the MF-GO enrichment analysis (Figure 3B), we detected a distinct biochemical specialization toward hydrolase activity. The most statistically significant terms were strictly associated with the cleavage of glycosidic bonds and the hydrolysis of O-glycosyl compounds, indicating a robust cellular capacity for complex carbohydrate processing. Concurrently, a significant enrichment of enzymatic functions associated with 1,3-beta-glucanosyltransferase and beta-glucosidase activities was observed, suggesting a pivotal role of vesicle proteins involved in the remodeling of glucans, which represent essential components of the fungal cell wall. Finally, CC-GO enrichment analysis (Figure 3C) further highlighted a strong prevalence of proteins associated with the fungal surface and external structures. The most statistically significant terms were “external encapsulating structure” and “fungal-type cell wall”, both exhibiting very high −log (FDR) values. Additionally, a significant enrichment was observed for the “extracellular region” and “cell periphery” categories, with the latter accounting for the highest gene count.

**Figure 4:**
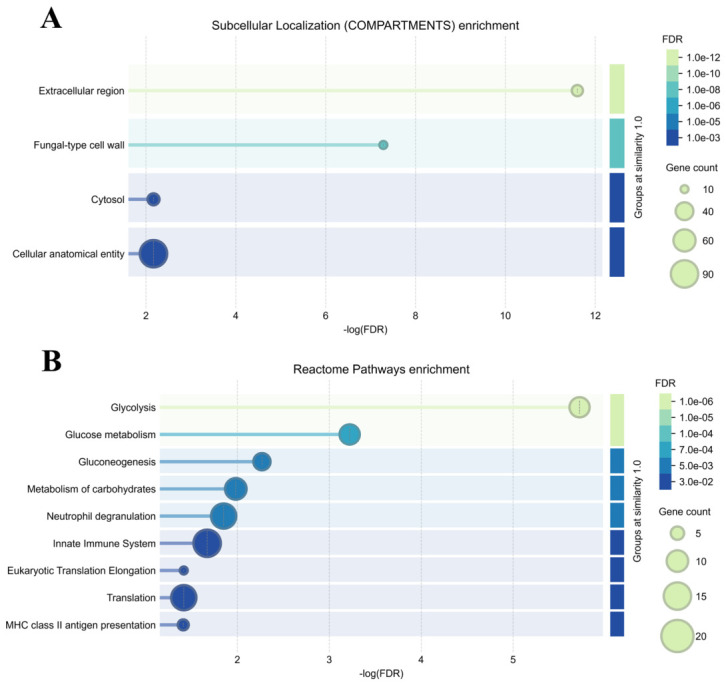
Subcellular localization (**A**) and Reactome (**B**) enrichment analysis of proteins identified in *K. marxianus* EVs. The figure shows the most significantly enriched terms based on false discovery rate (FDR) (color scale) and number of associated genes (bubble dimension).

The subcellular localization enrichment analysis (Figure 4A) demonstrated that the identified proteins are predominantly associated with compartments beyond the intracellular space. The most statistically significant terms were the extracellular region and the fungal-type cell wall, which exhibit the highest −log (FDR) values. Although the broad category “cellular anatomical entity” accounted for the largest number of proteins (gene count), the strong enrichment in cell wall and extracellular compartments further corroborated the extracellular nature of macromolecules and their functional specialization. The enrichment of the terms described above, along with the anchored component of the membrane category, suggests a possible origin of *K. marxianus* EVs. To identify enriched biological pathways, a Reactome-based functional analysis was further conducted, utilizing structural and functional homology to extrapolate the *K. marxianus* results to human pathways. The analysis highlighted a marked enrichment in proteins associated with energy metabolism—specifically glycolysis, glucose metabolism, and carbohydrate metabolism—as well as immune activation, including innate immunity, neutrophil degranulation, and MHC class II antigen presentation (Figure 4B). These results indicate that *K. marxianus* EVs are enriched with proteins essential for carbohydrate and glycoprotein modulation. Operating within the cell wall and extracellular environment, these proteins likely also contribute to host immune regulation, cell adhesion, and stress signaling pathways.

### 3.3. Safety Assessment of EVs In Vitro and In Vivo

**Figure 5:**
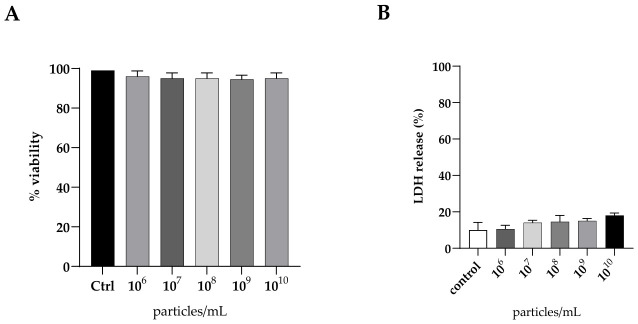
Cytotoxicity effect of *K. marxianus* EVs at concentration values of 10^6^, 10^7^, 10^8^,10^9^, and 10^10^ particles/mL on A-431 cells as measured with the MTT assay (**A**). Percentage of LDH release of A-431 cells treated with *K. marxianus* EVs at concentration values of 10^6^, 10^7^, 10^8^,10^9^, and 10^10^ particles/mL (**B**).

From the perspective of a potential use of EVs as postbiotics, it is mandatory to evaluate the safety of the preparation. Therefore, we tested the cytotoxicity of *K. marxianus* EVs in vitro using the vaginal epithelial A-431 cell line, measuring both the metabolic activity of the cells and the release of lactic dehydrogenase (LDH), an indicator of epithelial cell damage, into the surrounding medium after exposure to different EV densities (Figure 5). Both tests verified that EVs from *K. marxianus* had no toxicity *versus* the human cell model examined, since metabolic activity of cells exposed to EVs up to 10^10^ particles/mL remained unaltered with respect to control (Figure 5A), similar to the corresponding LDH release, which was comparable at all to the spontaneous release of the enzyme (Figure 5B).

**Figure 6:**
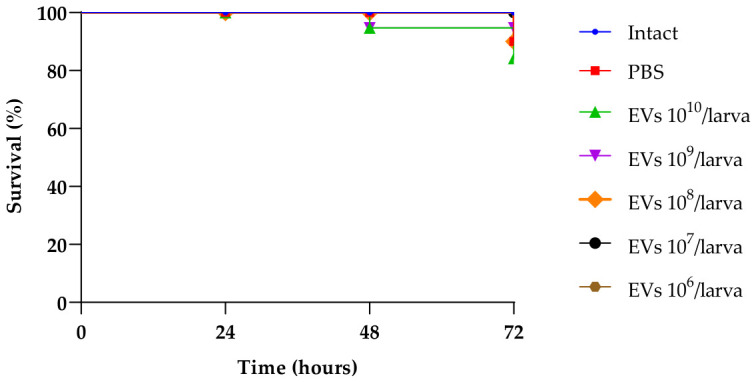
Kaplan–Meier survival curves of *Galleria mellonella* larvae injected with *K. marxianus* EVs at densities ranging from 10^6^ to 10^10^ particles per larva.

To test the safety of EVs in vivo, we used the commonly used animal model of *G. mellonella*. The larvae injected with EVs at densities ranging from 10^6^ to 10^10^ EVs per larva were monitored for 3 days; no significant loss of survival was observed in these cases when compared to the control group. A slight but statistically non-significant reduction in survival was observed only in the group of larvae injected with the highest density of EVs (10^10^ EVs per larva) (Figure 6).

### 3.4. EV Antagonistic Activity Versus C. albicans

**Figure 7:**
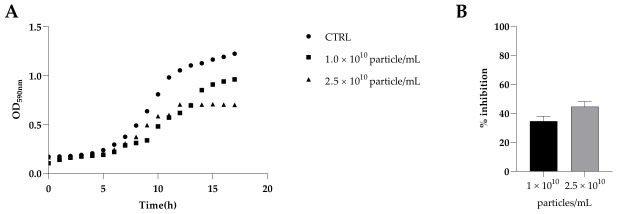
Growth curve of *C. albicans* C19 in the presence of *K. marxianus* EVs at a concentration of 1.3 × 10^10^ and 6 × 10^9^ particles/mL (**A**). Effect of *K. marxianus* EVs at a concentration of 1.3 × 10^10^ and 6.0 × 10^9^ particles/mL on *C. albicans* C19 biofilm formation (**B**).

**Figure 8:**
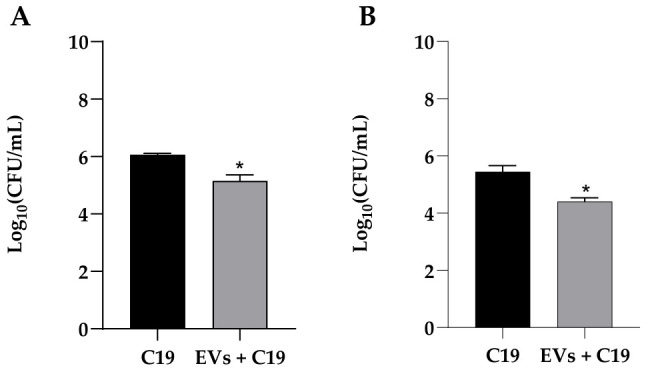
Effect of the *K. marxianus* EVs on adhesion (**A**) and invasion (**B**) of *C. albicans* C19 to A-431 epithelial cells. Data are presented as the mean ± SD of three independent experiments performed in triplicate. * *p* < 0.05 (t-test).

The antagonistic activity of *K. marxianus* EVs was evaluated by a series of experiments aiming at investigating the impact of these vesicles on some features, such as the growth of *C. albicans* in liquid culture and the pathogen biofilm formation in vitro (Figure 7), as well as adhesion and invasion in A-431 cells (Figure 8). For all the tests, we used the fluconazole-resistant clinical strain C19.

Growth analysis is a central feature to be examined in the evaluation of the antagonistic properties of a preparation. The results demonstrated that *K. marxianus* EVs can hinder the progression of the growth curve of *C. albicans* C19, and that the effect was dose-dependent (Figure 7A). The growth inhibition by EVs resulted in a lower amount of biomass at the end of the incubation period with the two EV doses employed (1.3 × 10^10^ and 6.0 × 10^9^ vesicles/mL, respectively), specifically 43 and 27% lower than that of the control.

The formation of *Candida* C19 biofilm in vitro was also examined in the presence of EVs at the same two concentration values used in the liquid cultures by determining the total yeast biomass of the C19 strain adhered to polystyrene in a conventional biofilm-forming test with crystal violet. In the presence of EVs, biofilm formation was impaired, with inhibitions of 42% and 37%, respectively, with respect to the control (Figure 7B).

We also tested the effect of the isolated EVs on the adhesion and invasiveness of *Candida* C19 in A-431 cells under conditions of pre-inoculation, where the putative postbiotic yeast EVs were added to the cell monolayer prior to the application of the *Candida* strain. The results of adhesion and invasiveness in the presence of *K. marxianus* EVs are reported in Figure 8, and in both cases, the effect of protection exerted by these vesicles was evident.

### 3.5. Immunomodulatory Effect

**Figure 9:**
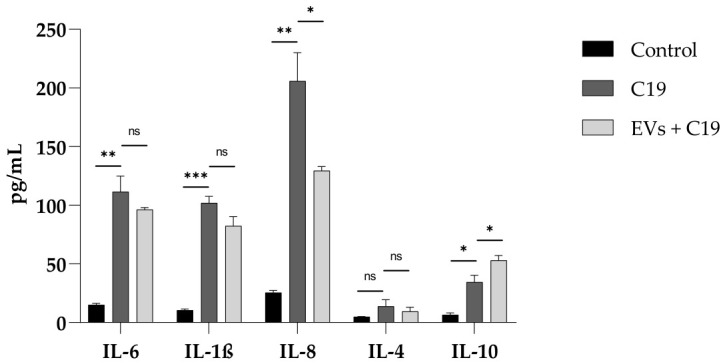
Cytokine modulation by *K. marxianus* EVs in A-431 infected with *C. albicans* C19. Data are presented as the mean ± SD of three independent experiments performed in triplicate. For each cytokine, C19 was compared with the untreated control, whereas EVs + C19 was compared with C19 (* = *p* < 0.05, ** = *p* < 0.01, *** = *p* < 0.001, ns = not significant; Dunnett’s test).

The possible immunomodulatory effect of *K. marxianus* EVs was analyzed by determining the concentration of pro-inflammatory (IL-6, IL-1β, and IL-8) and anti-inflammatory (IL-4 and IL-10) cytokines in the culture supernatants of A-431 treated with these vesicles before the infection with C19 and detected *via* ELISA. The infection with C19 determined *per se* an expected increase in IL-6, IL-1β, and IL-8 with respect to untreated cells (control), but it was significantly reduced when cells were pre-treated with *K. marxianus* EVs, especially in the case of IL-8, demonstrating the anti-inflammatory potential activity of these vesicles (Figure 9). Regarding the anti-inflammatory cytokines (IL-4 and IL-10), the effect of yeast EVs was evident in the case of IL-10, with a significant increase in IL-10 concentration, which was not observed in the case of IL-4 (Figure 9).

### 3.6. Expression of SAP2 and SAP6

**Figure 10:**
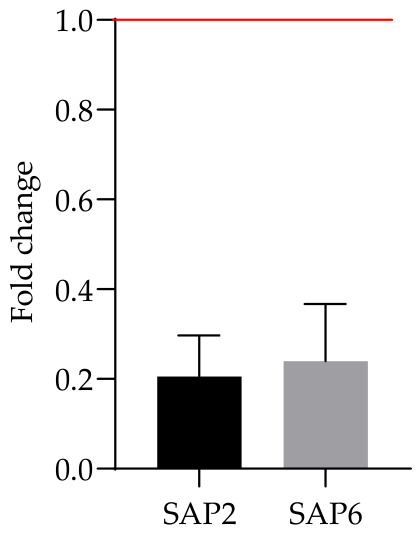
Effect of *K. marxianus* EVs on *SAP2* and *SAP6* gene expression in *Candida albicans* C19. Gene expression is reported as fold change relative to the untreated C19 control, which is set to 1 and indicated by the red reference line.

The expression of two genes associated with the complex phenomenon of invasion of the epithelial mucosa by *C. albicans*, namely, *SAP2* and *SAP6* coding for two aspartyl proteases, was investigated when C19 cells were co-incubated with *K. marxianus* EVs at a concentration of 1.6 × 10^8^ particles/mL. The results are shown in Figure 10, where the downregulation of the two genes examined in C19 cells following exposure to yeast EVs is shown.

## 4. Discussion

This work has started from the assumption that EVs from a probiotic microorganism can retain, at least in part, the beneficial features of the parent organism, representing a cell-free alternative to the use of live probiotic formulation. We recently isolated a probiotic *K. marxianus* strain whose extracellular isolate was assessed for its safety properties and antagonistic activity against some clinical fluconazole-resistant *C. albicans* strains [29]. On this basis, we have isolated a population of EVs from the liquid supernatant of this yeast strain and have characterized them. To our knowledge, this is the first time that the production of EVs by *K. marxianus* is reported. A 24 h culture was used to isolate the EV fraction, which is in agreement with the recent literature reporting that a larger amount of EVs per number of cells is secreted after 24 compared to 72 h cultivation [44]. Above-reported EVs had a diameter that was in the range reported for some populations of EVs from other yeast species, considering that different sizes generally reflect distinct mechanisms of biogenesis [40,42].

As reported above, the *K. marxianus* strain used in this work had previously been shown to exert an antagonistic activity against fluconazole-resistant *C. albicans* strains [29], enlarging the panel of probiotic yeasts that have been proposed as alternative or complementary therapeutic approaches in the case of VVC [45]. Indeed, *C. albicans*, the most common agent of human fungal infections, represents an increasing threat to public health due to the increasingly widespread resistance of clinical strains to conventional antifungals and to its low safety, so great efforts are being made to address alternative prophylactic and therapeutic approaches for candidiasis [46].

Within the fungal community, EVs are reported to be involved in several biological processes (cell-to-cell communication, response to nutrient availability, dimorphic transition, cell wall remodeling) or studied in interactions with the host [47]. In the case of probiotic yeasts, the potential antimicrobial activity of the secreted EVs has been scarcely explored so far. After assessing the complete safety of *K. marxianus* EVs both in vitro and in vivo, with the latter test carried out in the animal model *Galleria mellonella*, we have demonstrated that the exposure of *C. albicans* to these vesicles affects the progression of *Candida* cells in liquid culture. Treatment with *K. marxianus* EVs also influenced a typical trait associated with *Candida* virulence, which is the formation of biofilm, as well as a reduced expression of the genes *SAP2* and *SAP6*, coding for the most relevant aspartyl proteases involved in *Candida* infection. Our results also showed the inhibition of both adhesion and invasion of *C. albicans* to the vaginal epithelial cells of the A-431 cell line, as well as significant anti-inflammatory effects.

It is not easy to establish a cause-and-effect relationship in the observed phenomena, since EVs are a multi-component preparation that carries a diverse payload of proteins, nucleic acids, lipids, and other bioactive molecules. This complexity allows EVs to exert a wide range of effects, potentially through the synergistic interaction of their cargo components with various target (macro)molecules. In this work, we have focused on the proteins occurring in the isolated EVs. The results of a comprehensive proteomic analysis allowed for the identification of a total of 100 proteins, most of which (94) were highly interconnected, suggesting that most of the vesicular proteome operates through coordinated biological processes. The BP functional enrichment analysis of these assigned proteins revealed a dominance of components involved in central carbohydrate metabolism, especially concerning glycolysis and hexose catabolism. The occurrence of glycolytic proteins has already been reported in the cargo of EVs of several yeast species, including wine *S. cerevisiae* [22], as well as some probiotic strains of *S. boulardii* [21] and *Pichia kudriavzevii* [24]. Worth mentioning is the presence of glyceraldehyde-3-phosphate dehydrogenase (GAPDH) and enolase (accession codes A4ZGQ9 and W0T7K9 in Supplementary Table S1), since these proteins are known to be involved in adhesion processes [48] and vacuolar membrane fusion and protein transport [49], respectively, as a result of their ascertained moonlight nature, which confers them other functions beyond those associated with primary metabolism [50]. In addition, GAPDH is the source of antimicrobial peptides corresponding to proteolytic fragments of the parent protein secreted by *S. cerevisiae* during alcoholic fermentation, which are active against other yeasts and bacteria [51].

Furthermore, the MF enrichment analysis on the *K. marxianus* vesicular proteome evidenced the occurrence of various enzymes exerting a hydrolase activity on glycosidic bonds and O-glycosyl compounds, as well as various 1,3-beta-glucanosyltransferases and beta-glucosidases; a part of these components is involved in the remodeling of glucans, essential components of the fungal cell wall, together with chitin and various glycoproteins (Supplementary Table S1). In addition, a strong representation of extracellular proteins and components associated with the cell wall was also observed. These results align with those reported for EVs from other yeasts, which include various proteins involved in cell wall rearrangement [19,23,25], supporting *K. marxianus* as playing a pivotal role in EVs in cell wall dynamics. Specifically, among the enzymes involved in cell wall rearrangement, the occurrence of a chitinase and various glucanases (accession codes W0T491, W0TIA9, W0TI51, W0T898, W0T4U4, and W0T4Y9 in Supplementary Table S1) was relevant. These hydrolytic enzymes detected in the cell-free supernatant of *K. marxianus* have already been reported to act as antimicrobial compounds by inhibiting the growth of phytopathogenic fungi [52]. Similarly, cell wall-degrading enzymes, such as chitinases and proteases, secreted by other yeasts are thought to play an essential role in biocontrol [53].

A separate mention should be made in the case of ribosomal proteins (RPs), which were also detected in the cargo of the examined vesicles (accession codes W0T875, W0TA15, W0TDR9, P41770, W0TAQ3, W0T419, W0TCY5, and W0TBV5 in Supplementary Table S1). Evidence from eukaryotic systems suggests that these proteins can act as receptors that influence microbial pathogenesis [54]. On this basis, it is presumable that RPs in *K. marxianus* EVs might perform a moonlighting role, due to their recognized function as antimicrobial peptides, acting as vesicular toxins that provide a competitive edge on rival microbes in the extracellular environment [55].

The Reactome-based functional analysis conducted on the *K. marxianus* vesicular proteins highlighted a marked enrichment in proteins associated with immune activation, including innate immunity, neutrophil degranulation, and MHC class II antigen presentation, suggesting that a complex array of proteins may be involved in the immunomodulatory effects observed for these yeast EVs. It is well known that the interaction of fungal EVs with the host starts a process that can lead to the modulation of immune response, including the production of pro-inflammatory and anti-inflammatory cytokines [56], as also shown by a variety of in vitro assays [57].

In general, the mechanism of action of fungal EVs still has not been extensively studied so far, but presumably, it may resemble that reported for EVs from lactic acid bacteria. Therefore, also in the interaction of *K. marxianus* EVs with A-431 cells, a step of adhesion and subsequent internalization may be hypothesized, followed by modulation of cytokine secretion [58]. Further research will be aimed at elucidating the mechanisms of action of the cargo molecules of *K.marxianus* EVs with pattern recognition receptors and other surface molecules.

Moreover, from the perspective of a possible preventive and/or therapeutic use of *K. marxianus* EVs in VVC, it could be of interest to test the possible synergy of EVs in combination with conventional drugs as an adjunctive therapy to increase the antagonistic effect and reduce standard antifungal dosages (thereby minimizing toxicity).

Further investigations on in vivo models should be carried out, as well as the development of a proper formulation of the postbiotic preparation.

## 5. Conclusions

The presented results, though preliminary, show that EVs from *K. marxianus* may provide a cell-free alternative to probiotics, potentially representing an interesting postbiotic tool.

The secreted nanoparticles retain the biotherapeutic potential of the live probiotic: they inhibit *C. albicans* planktonic culture and biofilm formation, reduce the adhesion/invasion of the pathogen yeast on vaginal epithelial cells, downregulate the expression of the virulence-associated genes *SAP2* and *SAP6*, and reduce the inflammatory effects provoked by infection.

As inanimate particles, they are inherently non-viable, thus eliminating any risk of causing infections or transferring antibiotic resistance genes. Therefore, the move from the live organism to its EVs can be viewed as a re-contextualization of the probiotic action, positioning the microorganism not as the direct effector but as a biotechnological resource to produce these powerful nanoparticles.

## Data Availability

Proteomic data were deposited in the ProteomeXchange Consortium via the PRIDE partner repository with dataset identifier PXD078607.

## Supplementary Materials

The following supporting information can be downloaded at: https://www.mdpi.com/article/10.3390/pathogens15070667/s1, Supplementary Table S1. Detailed protein identifications of extracellular vesicles (EVs) derived from *Kluyveromyces marxianus* analyzed in this study. Reported are protein identification confidence (false discovery rate, FDR), protein accession number, gene name, protein description, experimental q-value, summed posterior error probability (PEP) score, sequence coverage (%), number of identified peptides, peptide spectrum matches (PSMs), number of unique peptides, protein length (amino acids, AAs), molecular mass (MW [kDa], Mascot identification score, and protein group assignment.
