# Supplementary material for: Extracellular Vesicles from Kluyveromyces marxianus as Potential Postbiotics Against Candida albicans Vaginal Infections
Source: Pathogens. 2026 Jun 25;15(7):667. doi: 10.3390/pathogens15070667 (PMC13414840; doi:10.3390/pathogens15070667)
Supplement: Supplementary file 1 [file pathogens-15-00667-s001.zip › pathogens-4368458-supplementary-1.pdf]

Table S1. Detailed protein identifications of extracellular vesicles (EVs) derived from Kluyveromyces marxianus analyzed in this study. Reported are protein identification confidence (false discovery rate, FDR), protein accession number, gene name, protein description, experimental q-value, summed posterior error probability (PEP) score, sequence coverage (%), number of identified peptides, peptide spectrum matches (PSMs), number of unique peptides, protein length (amino acids, AAs), molecular mass (MW [kDa], Mascot identification score, and protein group assignment.

Protein FDR Confidence	Accession	Gene Name	Description	Exp. q-value	Sum PEP Score	Coverage [%]	# Peptides	# PSMs	# Unique Peptides	# AAs	MW [kDa]	Score Mascot: Mascot	# Protein Groups
High	W0T9G4	DOG2	2-deoxyglucose-6-phosphate phosphatase 2 OS=Kluyveromyces marxianus (strain DMKU3-1042 / BCC 29191 / NBRC 104275) OX=1003335 GN=DOG2 PE=4 SV=1	0.005	2.547	5	1	2	1	248	27.2	84	1
High	W0T875	RPL27A	60S ribosomal protein L27-A OS=Kluyveromyces marxianus (strain DMKU3-1042 / BCC 29191 / NBRC 104275) OX=1003335 GN=RPL27A PE=3 SV=1	0.004	2.132	10	1	2	1	136	15.6	38	1
High	W0TA15	RPL6B	60S ribosomal protein L6-B OS=Kluyveromyces marxianus (strain DMKU3-1042 / BCC 29191 / NBRC 104275) OX=1003335 GN=RPL6B PE=3 SV=1	0.004	1.512	5	1	2	1	203	23.3	43	1
High	W0T8N1	SAH1	Adenosylhomocysteinase OS=Kluyveromyces marxianus (strain DMKU3-1042 / BCC 29191 / NBRC 104275) OX=1003335 GN=SAH1 PE=3 SV=1	0.004	1.744	3	1	2	1	449	49.2	60	1
High	Q07288	ADH1	Alcohol dehydrogenase 1 OS=Kluyveromyces marxianus OX=4911 GN=ADH1 PE=3 SV=1	0	3.379	7	2	2	1	348	37.1	67	1
High	G3FFC9	ADH1	alcohol dehydrogenase OS=Kluyveromyces marxianus OX=4911 GN=ADH1 PE=3 SV=1	0	3.574	7	2	2	1	348	37.1	66	1
High	W0TAR8	MNL1	alpha-1,2-Mannosidase OS=Kluyveromyces marxianus (strain DMKU3-1042 / BCC 29191 / NBRC 104275) OX=1003335 GN=MNL1 PE=3 SV=1	0.004	1.536	1	1	2	1	780	89.7	35	1
High	W0T9Q3	RAX2	Bud site selection protein RAX2 OS=Kluyveromyces marxianus (strain DMKU3-1042 / BCC 29191 / NBRC 104275) OX=1003335 GN=RAX2 PE=4 SV=1	0	4.236	1	1	2	1	1204	132.2	91	1
High	W0T9A7	DUG1	Cys-Gly metallodipeptidase DUG1 OS=Kluyveromyces marxianus (strain DMKU3-1042 / BCC 29191 / NBRC 104275) OX=1003335 GN=DUG1 PE=3 SV=1	0.004	1.558	2	1	2	1	518	57.5	43	1
High	W0T7M7	ASC1	Guanine nucleotide-binding protein subunit beta-like OS=Kluyveromyces marxianus (strain DMKU3-1042 / BCC 29191 / NBRC 104275) OX=1003335 GN=ASC1 PE=3 SV=2	0	3.412	4	1	2	1	326	35.7	81	1
High	W0TAF6	ECM14	Inactive metallocarboxypeptidase ECM14 OS=Kluyveromyces marxianus (strain DMKU3-1042 / BCC 29191 / NBRC 104275) OX=1003335 GN=ECM14 PE=3 SV=1	0	4.38	3	1	2	1	452	52.4	113	1
High	A4Z4U8	PPase	inorganic diphosphatase OS=Kluyveromyces marxianus OX=4911 GN=PPase PE=3 SV=1	0	3.078	5	1	2	1	287	32.2	69	1
High	W0TCB3	KEX1	KEX1 protease OS=Kluyveromyces marxianus (strain DMKU3-1042 / BCC 29191 / NBRC 104275) OX=1003335 GN=KEX1 PE=3 SV=1	0	3.69	2	1	2	1	775	86	73	1
High	W0TC44	TSA1	Peroxiredoxin TSA1 OS=Kluyveromyces marxianus (strain DMKU3-1042 / BCC 29191 / NBRC 104275) OX=1003335 GN=TSA1 PE=3 SV=1	0	4.369	7	1	2	1	197	21.6	80	1
High	W0TEV2	EMP47	Protein EMP47 OS=Kluyveromyces marxianus (strain DMKU3-1042 / BCC 29191 / NBRC 104275) OX=1003335 GN=EMP47 PE=4 SV=1	0	4.101	7	2	2	2	433	49.4	54	1
High	W0TDR9	ubi3	Ubiquitin-40S ribosomal protein S27a OS=Kluyveromyces marxianus (strain DMKU3-1042 / BCC 29191 / NBRC 104275) OX=1003335 GN=ubi3 PE=3 SV=1	0.004	2.18	4	1	2	1	151	17	68	1
High	W0TG88	ARF1	ADP-ribosylation factor OS=Kluyveromyces marxianus (strain DMKU3-1042 / BCC 29191 / NBRC 104275) OX=1003335 GN=ARF1 PE=3 SV=1	0	4.037	14	2	3	2	181	20.5	84	1
High	W0T6K4	EFT1	Elongation factor 2 OS=Kluyveromyces marxianus (strain DMKU3-1042 / BCC 29191 / NBRC 104275) OX=1003335 GN=EFT1 PE=4 SV=1	0	5.951	4	3	3	3	842	93.3	85	1
High	W0T6S4	ERO1	Endoplasmic oxidoreductin-1 OS=Kluyveromyces marxianus (strain DMKU3-1042 / BCC 29191 / NBRC 104275) OX=1003335 GN=ERO1 PE=3 SV=1	0	9.517	7	3	3	3	551	63.7	118	1
High	W0TAL8	GRP78	Endoplasmic reticulum chaperone BiP OS=Kluyveromyces marxianus (strain DMKU3-1042 / BCC 29191 / NBRC 104275) OX=1003335 GN=GRP78 PE=3 SV=1	0	7.715	7	3	3	3	679	74.3	90	1
High	W0TH78	TEF4	Eukaryotic elongation factor 1Bgamma OS=Kluyveromyces marxianus (strain DMKU3-1042 / BCC 29191 / NBRC 104275) OX=1003335 GN=TEF4 PE=4 SV=1	0	8.98	8	3	3	3	417	47.1	79	1
High	W0TF43	HARS	histidine--tRNA ligase OS=Kluyveromyces marxianus (strain DMKU3-1042 / BCC 29191 / NBRC 104275) OX=1003335 GN=HARS PE=3 SV=1	0.004	2.18	2	1	3	1	519	57.6	58	1

High	Q6I7B7	oye	Old yellow enzyme OS=Kluyveromyces marxianus OX=4911 GN=oye PE=1 SV=1	0	6.163	9	3	3	3	403	45.9	89	1
High	W0TE22	SEC53	Phosphomannomutase OS=Kluyveromyces marxianus (strain DMKU3-1042 / BCC 29191 / NBRC 104275) OX=1003335 GN=SEC53 PE=3 SV=1	0	3.506	9	2	3	2	254	29	97	1
High	W0TA42	AXL2	Protein AXL2 OS=Kluyveromyces marxianus (strain DMKU3-1042 / BCC 29191 / NBRC 104275) OX=1003335 GN=AXL2 PE=4 SV=1	0	5.169	4	2	3	2	785	86.7	144	1
High	P41770	SSB1	Ribosome-associated molecular chaperone SSB1 OS=Kluyveromyces marxianus OX=4911 GN=SSB1 PE=3 SV=2	0	4.006	4	2	3	2	613	66	84	1
High	W0T4F1	SAM2	S-adenosylmethionine synthase OS=Kluyveromyces marxianus (strain DMKU3-1042 / BCC 29191 / NBRC 104275) OX=1003335 GN=SAM2 PE=3 SV=1	0	2.892	4	1	3	1	384	42.3	70	1
High	W0T6G4	PRY2	SCP super family OS=Kluyveromyces marxianus (strain DMKU3-1042 / BCC 29191 / NBRC 104275) OX=1003335 GN=PRY2 PE=4 SV=1	0	3.606	3	1	3	1	363	37.7	87	1
High	W0T3V9	GND1	6-phosphogluconate dehydrogenase, decarboxylating OS=Kluyveromyces marxianus (strain DMKU3-1042 / BCC 29191 / NBRC 104275) OX=1003335 GN=GND1 PE=3 SV=1	0	6.072	6	2	4	2	492	53.4	141	1
High	W0T6N5	PHO12	acid phosphatase OS=Kluyveromyces marxianus (strain DMKU3-1042 / BCC 29191 / NBRC 104275) OX=1003335 GN=PHO12 PE=3 SV=1	0	4.37	4	2	4	2	482	54.5	98	1
High	A1IIA4	KmADH4	alcohol dehydrogenase OS=Kluyveromyces marxianus OX=4911 GN=KmADH4 PE=3 SV=1	0	6.455	7	3	4	1	379	40.4	97	1
High	W0TBU9	BAR1	Aspartic proteinase yapsin-3 OS=Kluyveromyces marxianus (strain DMKU3-1042 / BCC 29191 / NBRC 104275) OX=1003335 GN=BAR1 PE=3 SV=1	0	15.291	11	3	4	3	501	54.7	166	1
High	W0TD70	HSP82	ATP-dependent molecular chaperone HSC82 OS=Kluyveromyces marxianus (strain DMKU3-1042 / BCC 29191 / NBRC 104275) OX=1003335 GN=HSP82 PE=3 SV=1	0	6.895	6	3	4	3	713	81.4	94	1
High	W0TEY2	KLMA_70062	Covalently-linked cell wall protein 14 OS=Kluyveromyces marxianus (strain DMKU3-1042 / BCC 29191 / NBRC 104275) OX=1003335 GN=KLMA_70062 PE=4 SV=1	0	7.228	9	1	4	1	228	21.2	330	1
High	W0T4R5	RAG2	Glucose-6-phosphate isomerase OS=Kluyveromyces marxianus (strain DMKU3-1042 / BCC 29191 / NBRC 104275) OX=1003335 GN=RAG2 PE=3 SV=1	0	10.544	6	3	4	3	555	61.6	161	1
High	W0TCS3	AIM2	Protein AIM2 OS=Kluyveromyces marxianus (strain DMKU3-1042 / BCC 29191 / NBRC 104275) OX=1003335 GN=AIM2 PE=4 SV=1	0	8.736	15	2	4	2	253	27.6	203	1
High	W0T962	HOC1	Putative glycosyltransferase HOC1 OS=Kluyveromyces marxianus (strain DMKU3-1042 / BCC 29191 / NBRC 104275) OX=1003335 GN=HOC1 PE=3 SV=1	0	4.841	5	2	4	2	403	46.3	92	1
High	W0TAK9	MSB2	Hansenula MRAKII killer toxin-resistant protein OS=Kluyveromyces marxianus (strain DMKU3-1042 / BCC 29191 / NBRC 104275) OX=1003335 GN=MSB2 PE=4 SV=1	0	6.548	2	1	5	1	866	89	172	1
High	W0T3H5	PGK	Phosphoglycerate kinase OS=Kluyveromyces marxianus (strain DMKU3-1042 / BCC 29191 / NBRC 104275) OX=1003335 GN=PGK PE=3 SV=1	0	13.609	13	4	5	4	416	44.4	149	1
High	W0T812	PRY2	Protein PRY1 OS=Kluyveromyces marxianus (strain DMKU3-1042 / BCC 29191 / NBRC 104275) OX=1003335 GN=PRY2 PE=4 SV=1	0	7.685	10	2	5	2	305	31.1	117	1
High	W0TGU0	UTH1	Protein UTH1 OS=Kluyveromyces marxianus (strain DMKU3-1042 / BCC 29191 / NBRC 104275) OX=1003335 GN=UTH1 PE=3 SV=1	0	4.406	6	3	5	3	381	39.2	101	1
High	W0TAQ3	RPS13	40S ribosomal protein S13 OS=Kluyveromyces marxianus (strain DMKU3-1042 / BCC 29191 / NBRC 104275) OX=1003335 GN=RPS13 PE=3 SV=1	0.004	1.567	8	1	6	1	151	17	148	1
High	W0T419	RPS16	40S ribosomal protein S16 OS=Kluyveromyces marxianus (strain DMKU3-1042 / BCC 29191 / NBRC 104275) OX=1003335 GN=RPS16 PE=3 SV=1	0	2.69	10	1	6	1	143	15.9	157	1
High	W0T5M4	PRC1	Carboxypeptidase OS=Kluyveromyces marxianus (strain DMKU3-1042 / BCC 29191 / NBRC 104275) OX=1003335 GN=PRC1 PE=3 SV=1	0	15.578	12	5	6	5	540	60.8	171	1
High	W0THY4	KNH1	Cell wall synthesis protein KNH1 OS=Kluyveromyces marxianus (strain DMKU3-1042 / BCC 29191 / NBRC 104275) OX=1003335 GN=KNH1 PE=3 SV=1	0	7.715	12	2	6	2	275	29.6	146	1
High	W0THY0	SSA3	Heat shock protein SSA3 OS=Kluyveromyces marxianus (strain DMKU3-1042 / BCC 29191 / NBRC 104275) OX=1003335 GN=SSA3 PE=3 SV=1	0	9.26	10	5	6	5	650	70	102	1
High	W0TBW0	PAB1	Polyadenylate-binding protein OS=Kluyveromyces marxianus (strain DMKU3-1042 / BCC 29191 / NBRC 104275) OX=1003335 GN=PAB1 PE=3 SV=1	0.004	1.826	2	1	6	1	590	65.9	124	1
High	W0T3J8	TIF1	RNA helicase OS=Kluyveromyces marxianus (strain DMKU3-1042 / BCC 29191 / NBRC 104275) OX=1003335 GN=TIF1 PE=3 SV=1	0	3.385	9	3	6	3	396	44.6	101	1
High	W0T257	PRD1	Saccharolysin OS=Kluyveromyces marxianus (strain DMKU3-1042 / BCC 29191 / NBRC 104275) OX=1003335 GN=PRD1 PE=3 SV=1	0	11.057	6	4	6	4	712	82.3	150	1

High	W0TFQ2	PEP4	Saccharopepsin OS=Kluyveromyces marxianus (strain DMKU3-1042 / BCC 29191 / NBRC 104275) OX=1003335 GN=PEP4 PE=3 SV=1	0	12.694	11	3	6	3	408	44.1	240	1
High	Q70JN8	TPI1	Triosephosphate isomerase OS=Kluyveromyces marxianus OX=4911 GN=TPI1 PE=3 SV=1	0	7.655	22	4	6	4	248	26.9	122	1
High	W0TCY5	RPL12B	60S ribosomal protein L12 OS=Kluyveromyces marxianus (strain DMKU3-1042 / BCC 29191 / NBRC 104275) OX=1003335 GN=RPL12B PE=3 SV=1	0	3.701	7	1	8	1	165	17.8	354	1
High	W0T9W3	FBA1	Fructose-bisphosphate aldolase OS=Kluyveromyces marxianus (strain DMKU3-1042 / BCC 29191 / NBRC 104275) OX=1003335 GN=FBA1 PE=3 SV=1	0	10.289	16	4	8	4	361	39.5	172	1
High	W0TIA9	KLMA_70011	glucan endo-1,3-beta-D-glucosidase OS=Kluyveromyces marxianus (strain DMKU3-1042 / BCC 29191 / NBRC 104275) OX=1003335 GN=KLMA_70011 PE=3 SV=1	0	8.989	7	2	8	2	395	43.1	267	1
High	P84998	GAP1	Glyceraldehyde-3-phosphate dehydrogenase 1 OS=Kluyveromyces marxianus OX=4911 GN=GAP1 PE=1 SV=1	0	10.573	12	4	8	2	329	35.2	248	1
High	W0T511	HTZ1	Histone H2A OS=Kluyveromyces marxianus (strain DMKU3-1042 / BCC 29191 / NBRC 104275) OX=1003335 GN=HTZ1 PE=3 SV=1	0	4.827	7	1	8	1	132	14.1	213	1
High	W0T333	KRE9	Cell wall synthesis protein KRE9 OS=Kluyveromyces marxianus (strain DMKU3-1042 / BCC 29191 / NBRC 104275) OX=1003335 GN=KRE9 PE=3 SV=1	0	17.695	29	4	9	4	274	30.9	338	1
High	W0T6W1	GPM1	Phosphoglycerate mutase OS=Kluyveromyces marxianus (strain DMKU3-1042 / BCC 29191 / NBRC 104275) OX=1003335 GN=GPM1 PE=3 SV=2	0	9.325	15	3	9	3	260	28.8	248	1
High	P33149	PDC1	Pyruvate decarboxylase OS=Kluyveromyces marxianus OX=4911 GN=PDC1 PE=3 SV=1	0	6.611	8	3	9	3	564	61.9	227	1
High	W0TKF9	MET6	5-methyltetrahydropteroyltriglutamate--homocysteine S-methyltransferase OS=Kluyveromyces marxianus (strain DMKU3-1042 / BCC 29191 / NBRC 104275) OX=1003335 GN=MET6 PE=3 SV=1	0	11.594	11	7	10	7	768	86.3	178	1
High	W0TIE1	ACT	Actin OS=Kluyveromyces marxianus (strain DMKU3-1042 / BCC 29191 / NBRC 104275) OX=1003335 GN=ACT PE=3 SV=1	0	10.193	9	3	10	3	378	42	275	1
High	W0T7Q2	EGT2	Uncharacterized protein OS=Kluyveromyces marxianus (strain DMKU3-1042 / BCC 29191 / NBRC 104275) OX=1003335 GN=EGT2 PE=4 SV=1	0	3.374	1	1	10	1	575	60.8	343	1
High	W0T598	BMH2	Protein BMH2 OS=Kluyveromyces marxianus (strain DMKU3-1042 / BCC 29191 / NBRC 104275) OX=1003335 GN=BMH2 PE=3 SV=1	0	8.45	21	4	11	4	250	28.3	202	1
High	W0T2Y2	GAS3	1,3-beta-glucanosyltransferase OS=Kluyveromyces marxianus (strain DMKU3-1042 / BCC 29191 / NBRC 104275) OX=1003335 GN=GAS3 PE=3 SV=1	0	19.484	13	6	12	6	503	54.5	374	1
High	W0TBV5	RPL2	60S ribosomal protein L2 OS=Kluyveromyces marxianus (strain DMKU3-1042 / BCC 29191 / NBRC 104275) OX=1003335 GN=RPL2 PE=3 SV=1	0	2.679	4	1	12	1	254	27.4	292	1
High	W0TFN7	PIR3	Cell wall mannoprotein HSP150 OS=Kluyveromyces marxianus (strain DMKU3-1042 / BCC 29191 / NBRC 104275) OX=1003335 GN=PIR3 PE=3 SV=1	0	6.322	5	2	13	2	342	34.6	272	1
High	W0T5V4	YPS1	Aspartic proteinase 3 OS=Kluyveromyces marxianus (strain DMKU3-1042 / BCC 29191 / NBRC 104275) OX=1003335 GN=YPS1 PE=3 SV=1	0	25.236	15	7	14	7	601	63.1	419	1
High	W0TGV8	TEF	Elongation factor 1-alpha OS=Kluyveromyces marxianus (strain DMKU3-1042 / BCC 29191 / NBRC 104275) OX=1003335 GN=TEF PE=3 SV=1	0	8.536	7	3	14	3	458	49.9	309	1
High	W0TDC4	MUC1	Flo11 super family OS=Kluyveromyces marxianus (strain DMKU3-1042 / BCC 29191 / NBRC 104275) OX=1003335 GN=MUC1 PE=4 SV=1	0	22.546	9	4	16	4	874	87.3	435	1
High	W0TF03	AGP1	High-affinity glutamine permease OS=Kluyveromyces marxianus (strain DMKU3-1042 / BCC 29191 / NBRC 104275) OX=1003335 GN=AGP1 PE=4 SV=1	0.004	2.072	1	1	16	1	581	63.6	288	1
High	W0TDT2	PYK1	Pyruvate kinase OS=Kluyveromyces marxianus (strain DMKU3-1042 / BCC 29191 / NBRC 104275) OX=1003335 GN=PYK1 PE=3 SV=1	0	12.341	17	7	16	7	501	54.8	261	1
High	Q9P4C2	ADH2	Alcohol dehydrogenase 2 OS=Kluyveromyces marxianus OX=4911 GN=ADH2 PE=3 SV=3	0	22.385	24	8	17	6	348	36.9	441	1
High	A4ZGQ9	GAPDH	Glyceraldehyde-3-phosphate dehydrogenase OS=Kluyveromyces marxianus OX=4911 GN=GAPDH PE=3 SV=1	0	21.707	36	8	17	6	331	35.5	415	1
High	W0T2S9	PLB	Lysophospholipase OS=Kluyveromyces marxianus (strain DMKU3-1042 / BCC 29191 / NBRC 104275) OX=1003335 GN=PLB PE=3 SV=1	0	22.076	12	7	22	7	652	71.2	532	1
High	W0T6N1	KLMA_10829	Cell wall protein CWP1 OS=Kluyveromyces marxianus (strain DMKU3-1042 / BCC 29191 / NBRC 104275) OX=1003335 GN=KLMA_10829 PE=4 SV=1	0	21.328	22	4	23	4	319	32.9	526	1
High	A0A1V0FZV4	HXK1	Phosphotransferase OS=Kluyveromyces marxianus OX=4911 GN=HXK1 PE=3 SV=1	0	21.12	18	6	24	6	485	53.7	646	1

High	W0TFU2	GAS1	1,3-beta-glucanosyltransferase OS=Kluyveromyces marxianus (strain DMKU3-1042 / BCC 29191 / NBRC 104275) OX=1003335 GN=GAS1 PE=3 SV=1	0	33.018	21	9	26	9	568	60.4	763	1
High	W0TCC7	GAS5	1,3-beta-glucanosyltransferase OS=Kluyveromyces marxianus (strain DMKU3-1042 / BCC 29191 / NBRC 104275) OX=1003335 GN=GAS5 PE=3 SV=1	0	19.062	16	5	26	5	487	51.8	1091	1
High	W0TFJ8	CWP1	Cell wall protein CWP1 OS=Kluyveromyces marxianus (strain DMKU3-1042 / BCC 29191 / NBRC 104275) OX=1003335 GN=CWP1 PE=4 SV=1	0	21.369	28	4	27	4	259	25.6	746	1
High	W0TCN1	ECM33	Cell wall protein ECM33 OS=Kluyveromyces marxianus (strain DMKU3-1042 / BCC 29191 / NBRC 104275) OX=1003335 GN=ECM33 PE=3 SV=1	0	27.462	23	6	29	6	420	42.7	850	1
High	W0T5X0	KLMA_10826	Uncharacterized protein OS=Kluyveromyces marxianus (strain DMKU3-1042 / BCC 29191 / NBRC 104275) OX=1003335 GN=KLMA_10826 PE=4 SV=1	0	13.788	13	2	33	2	220	22.8	827	1
High	W0T491	CTS1	chitinase OS=Kluyveromyces marxianus (strain DMKU3-1042 / BCC 29191 / NBRC 104275) OX=1003335 GN=CTS1 PE=3 SV=1	0	53.021	31	10	34	10	556	58.9	1233	1
High	W0TA48	SCW4	Probable family 17 glucosidase SCW10 OS=Kluyveromyces marxianus (strain DMKU3-1042 / BCC 29191 / NBRC 104275) OX=1003335 GN=SCW4 PE=3 SV=2	0	21.418	21	6	34	6	405	42.7	974	1
High	W0TI51	BGL2	glucan 1,3-beta-glucosidase OS=Kluyveromyces marxianus (strain DMKU3-1042 / BCC 29191 / NBRC 104275) OX=1003335 GN=BGL2 PE=3 SV=1	0	16.326	17	4	37	4	312	34	1005	1
High	W0TC08	CRH1	Glycosidase OS=Kluyveromyces marxianus (strain DMKU3-1042 / BCC 29191 / NBRC 104275) OX=1003335 GN=CRH1 PE=3 SV=1	0	33.694	18	6	37	6	459	47.8	1330	1
High	W0T6Q2	PHO3	acid phosphatase OS=Kluyveromyces marxianus (strain DMKU3-1042 / BCC 29191 / NBRC 104275) OX=1003335 GN=PHO3 PE=3 SV=1	0	34.858	23	8	38	8	471	53.8	913	1
High	W0T4H4	SCW11	Probable family 17 glucosidase SCW11 OS=Kluyveromyces marxianus (strain DMKU3-1042 / BCC 29191 / NBRC 104275) OX=1003335 GN=SCW11 PE=3 SV=1	0	37.689	19	6	42	6	518	53.2	1433	1
High	W0T946	UTR2	Glycosidase OS=Kluyveromyces marxianus (strain DMKU3-1042 / BCC 29191 / NBRC 104275) OX=1003335 GN=UTR2 PE=3 SV=1	0	30.928	16	6	45	6	470	50.6	1433	1
High	W0T7K9	ENO	phosphopyruvate hydratase OS=Kluyveromyces marxianus (strain DMKU3-1042 / BCC 29191 / NBRC 104275) OX=1003335 GN=ENO PE=3 SV=1	0	53.873	43	12	63	12	437	46.8	1911	1
High	W0T504	PGU1	endo-polygalacturonase OS=Kluyveromyces marxianus (strain DMKU3-1042 / BCC 29191 / NBRC 104275) OX=1003335 GN=PGU1 PE=3 SV=2	0	72.632	36	10	66	10	394	41.2	1891	1
High	W0T4X9	KLMA_10833	Uncharacterized protein C800.11 OS=Kluyveromyces marxianus (strain DMKU3-1042 / BCC 29191 / NBRC 104275) OX=1003335 GN=KLMA_10833 PE=3 SV=1	0	51.99	31	9	83	9	368	40.4	3050	1
High	W0T898	TOS1	glucan endo-1,3-beta-D-glucosidase OS=Kluyveromyces marxianus (strain DMKU3-1042 / BCC 29191 / NBRC 104275) OX=1003335 GN=TOS1 PE=3 SV=1	0	57.958	34	9	85	9	462	47.7	2868	1
High	W0TI90	SIM1	Septation protein SUN4 OS=Kluyveromyces marxianus (strain DMKU3-1042 / BCC 29191 / NBRC 104275) OX=1003335 GN=SIM1 PE=3 SV=1	0	71.863	25	8	109	8	403	41.8	4154	1
High	W0TK22	SCW4	Probable family 17 glucosidase SCW4 OS=Kluyveromyces marxianus (strain DMKU3-1042 / BCC 29191 / NBRC 104275) OX=1003335 GN=SCW4 PE=3 SV=1	0	57.163	23	7	120	7	377	39.1	4024	1
High	W0T4U4	DSE4	glucan endo-1,3-beta-D-glucosidase OS=Kluyveromyces marxianus (strain DMKU3-1042 / BCC 29191 / NBRC 104275) OX=1003335 GN=DSE4 PE=3 SV=1	0	159.064	38	20	142	20	1011	108.9	4484	1
High	W0TD05	PIR1	Cell wall mannoprotein HSP150 OS=Kluyveromyces marxianus (strain DMKU3-1042 / BCC 29191 / NBRC 104275) OX=1003335 GN=PIR1 PE=3 SV=1	0	37.819	11	4	155	1	391	39.4	7817	1
High	W0TBA3	PIR1	Cell wall mannoprotein HSP150 OS=Kluyveromyces marxianus (strain DMKU3-1042 / BCC 29191 / NBRC 104275) OX=1003335 GN=PIR1 PE=3 SV=1	0	40.891	19	5	165	2	321	32.3	8077	1
High	W0T4Y9	KLMA_10234	glucan 1,3-beta-glucosidase OS=Kluyveromyces marxianus (strain DMKU3-1042 / BCC 29191 / NBRC 104275) OX=1003335 GN=KLMA_10234 PE=3 SV=1	0	139.746	55	21	290	21	431	49.5	7984	1
High	P28999	INU1	Inulinase OS=Kluyveromyces marxianus OX=4911 GN=INU1 PE=1 SV=1	0	175.274	54	19	1166	2	555	62.2	43,448	1
High	W0T408	INU1	Inulinase OS=Kluyveromyces marxianus (strain DMKU3-1042 / BCC 29191 / NBRC 104275) OX=1003335 GN=INU1 PE=1 SV=1	0	182.711	48	18	1170	1	556	62.3	43,701	1
